# Synergistic effect of mesoporous silica nanocarrier-assisted photodynamic therapy and anticancer agent activity on lung cancer cells

**DOI:** 10.1007/s10103-023-03969-x

**Published:** 2024-03-16

**Authors:** Burcu Güleryüz, Ayşe Işık, Murat Gülsoy

**Affiliations:** 1 Institute of Biomedical Engineering, Bogazici University, Uskudar, Istanbul, 34684 Turkey; 2 Department of Molecular Biology and Genetics, Halic University, Eyupsultan, Istanbul, 34060 Turkey

**Keywords:** Photodynamic Therapy, Anticancer Therapy, Combination Therapy, Nanomedicine, Lung Cancer

## Abstract

Investigating combined treatment methodologies is crucial for addressing the complex nature of cancer. As an emerging strategy, nano-biotechnology encourages the design of unique nanocarriers possessing simultaneous therapeutic application properties. This study aims to explore the combined effects of photodynamic and anticancer treatments using a multifunctional nanocarrier system co-administering the photosensitizer IR780 and the anticancer agent curcumin (Cur) on lung cancer cells. Nanocarriers were prepared by encapsulation IR780 and Cur inside polyethylene glycol-capped mesoporous silica nanoparticles (Cur&IR780@MSN). Various concentrations of nanocarriers were evaluated on A549 cells following 5 min NIR laser light (continuous wave, 785 nm, 500 mW/cm^2^) irradiation. The internalization of nanocarriers was observed through the fluorescence of Cur. Changes in cell viability were determined using the MTT assay and AO/PI staining. A scratch assay analysis was also performed to examine the impact of combined treatments on cell migration. Characterization of the nanocarriers revealed adequate hydrophobic drug loading, temperature-inhibited feature, enhanced reactive oxygen species generation, a pH-dependent curcumin release profile, and high biocompatibility. Cur&IR780@MSN, which enabled the observation of synergistic treatment efficacy, successfully reduced cell viability by up to 78%. In contrast, monotherapies with curcumin-loaded nanocarriers (Cur@MSN) and IR780-loaded nanocarriers (IR780@MSN) resulted in a 38% and 56% decrease in cell viability, respectively. The constructed Cur&IR780@MSN nanocarrier has demonstrated remarkable performance in the application of combination therapies for lung cancer cells. These nanocarriers have the potential to inspire future studies in tumor treatment methods.

**Supplementary Information:**

The online version contains supplementary material available at 10.1007/s10103-023-03969-x.

## Introduction

Conventional cancer treatment approaches, such as chemotherapy, radiotherapy, and surgical dissection are insufficient [1]. In order to overcome the complex environment of cancer diseases, it is crucial to employ treatments with site-specific features, minimal side-effects on healthy tissue, and strategies that prevents metastasis [2, 3]. The most promising approach to meet these requirements simultaneously is to use nanotechnology with safe and controllable therapeutic techniques (i.e. photodynamic, photothermal or anticancer-drug therapies) [4–6].

Photodynamic therapy (PDT) is an emerging and minimally invasive tumor treatment methodology [7]. Its therapeutic mechanism can be explained as the conjugation of photosensitizers (PS) and light to generate reactive oxygen species (ROS) in the presence of oxygen [8]. When PS is activated with an appropriate wavelength, ROS induces local cytotoxicity and eventually death of tumor cells. The effectiveness of PDT relies mainly on (I) the type and concentration of administered PS; (II) the wavelength, power and duration of the applied light; and (III) the endogenous oxygen level [9–11]. Therefore, it is necessary to provide optimal conditions with the selected PS and applied light for successful implementation of PDT.

Among many commercially available photosensitizers, near-infrared (NIR) light triggered dyes (i.e., IR780, IR783, IR820, IR825, and indocyanine green (ICG)) have better penetration depth ability than the visible-range activated PS [12–16]. Their potential use has been reported in previous studies [17]. For instance, our group analyzed the U.S. Food and Drug Administration (FDA)-approved ICG in prostate (PC-3), colon (Caco-2) and neuroblastoma (SH-SY5Y) cancer cells but low singlet oxygen (^1^O_2_) quantum yield (QY) problem of ICG was limited its performance as a PDT agent [18]. When considering NIR probes, IR780 possess distinct advantageous over ICG such as more stable structure, better fluorescence properties, selective accumulation behavior in the mitochondria of cancer cells and higher singlet oxygen quantum yield (^1^O_2_ QY of IR780: 0.127 vs. ^1^O_2_ QY of ICG: 0.002) [19]. Nevertheless, the fact remains that complete eradication of tumors with photodynamic therapy is not possible due to the emergence of resistance against PDT in cancer cells. Combining PDT with other safe anti-cancer therapies can be a solution to this limitation.

Various natural products are valuable candidates as anti-cancer agents since they may offer harmless chemopreventive and chemotherapeutic activities [6]. Over the years, naturally occurring curcumin (Cur), the major ingredient of the turmeric (*Curcuma longa*), has drawn great attention for its therapeutic and pharmacologic effects [20, 21]. Cur has the ability to inhibit proliferation and invasion, induce cancer cell death mechanisms, and suppress the metastasis of tumor, through numerous cell signaling pathways [22, 23]. For example, Cur successfully inhibited non-small lung cancer cells by regulating of metalloproteinase (MMP)-9, epidermal growth factor receptor (EGFR), drug-efflux protein P-gp, and Bcl-2/Bax protein [24]. Researches have also shown that possible PDT resistance due to nitric oxide generation, over-expressed mRNA levels of EGFR, or upregulation of anti-apoptotic Bcl-2 family proteins in tumor cells can be eliminated by the administration of Cur [25–27]. In spite of the favorable cancer treatment activities of Cur and PDT agents, their poor pharmacokinetic and pharmacodynamic properties limits the free curcumin and photosensitizer usage [25]. Based on these, the application of combined treatments via Cur&PS necessitates the utilization of a nanocarrier delivery system for overcoming both the resistance proteins (i.e., Pg-p) to foreign compounds and the low bioavailability of the agents at the tumor site [23, 27].

Mesoporous silica nanoparticles (MSN) have long been a focus of research in nanotechnology as a drug carriers for cancer cells due to their low toxicity, high surface area-to-volume ratio, thermal and chemical stability, as well as ease surface functionalization [28]. In addition, MSN can simultaneously encapsulate various hydrophilic/hydrophobic drugs and protect the therapeutic molecules from degradation thereby enhancing their bioavailability. Besides, the drug delivery properties of MSN can be improved through appropriate surface modification [29]. Notably, the functionalization of MSN with polyethylene glycol (PEG) extends the blood circulation time of nanocarriers, prevents early leakage of agents, and supports pH-dependent drug release [30]. PEG-coated MSN can successfully accumulate in cancer cells through the enhanced permeability and retention effect, thus; drugs encapsulated within the silica pores hold promising potential for exerting superior therapeutic effect.

In this study, PEG-functionalized mesoporous silica nanocarriers (MSN) were synthesized to deliver IR780 and Cur to tumor cells simultaneously. It was aimed to investigate the synergistic effects of photodynamic and anticancer therapies using these engineered nanocarriers on lung cancer cells (A549). The developed combination treatment strategy exhibited excellent outcomes when compared to monotherapies involving PDT or anticancer therapy.

## Materials and Methods

### Materials

Ammonium hydroxide (NH_4_OH), cetyltrimethylammonium bromide (CTAB), tetraethyl orthosilicate (TEOS), hydrochloric acid (HCl), curcumin, IR780, polyethylene glycol (PEG-4000) 1,3-Diphenylisobenzofuran (DPBF), Dimethyl sulfoxide (DMSO), 3-(4,5-dimethylthiazol-2-yl)-2,5-diphenyl-trazoliumbromide (MTT), singlet oxygen sensor green (SOSG), acridine orange (AO), and propidium iodide (PI) were purchased from Sigma-Aldrich. RPMI 1640 medium, fetal bovine serum (FBS), and penicillin–streptomycin solution were acquired from Biosera.

### Mesoporous Silica Synthesis

The production of mesoporous silica nanoparticles was achieved by some adjustments in the synthesis protocol presented in the literature [31]. Firstly, 0.5 M NH_4_OH solution was prepared with distilled water. Then, 0.058 g of CTAB was added and mixed with NH_4_OH solution at 500 RPM under 40 ˚C. 600 µL of ethanol-TEOS (0.88 M) were poured dropwise for precipitation of nanoparticles. The temperature of the mixture was kept at 60 ˚C for two hours. Afterward, the solution was centrifuged (14,800 RPM-15 min) to collect the nanoparticles and remove the excess chemicals. The washing procedure was applied three times with ethanol. Note that, ultrasonic probe (Omni-Ruptor 4000) was used to disperse the nanoparticles. MSN synthesis was completed after the CTAB removal. 20 µL hydrochloric acid and nanoparticles in 20 mL ethanol were mixed at 60 ˚C for three hours. Then, nanoparticles were washed with ethanol. At the end of the production protocol, the amount of MSN was around 15 mg.

### Curcumin and IR780 Loading

Three different types of nanocarrier were developed; curcumin loaded nanocarrier (Cur@MSN), IR780 loaded nanocarrier (IR780@MSN), and curcumin-IR780 loaded nanocarrier (Cur&IR780@MSN). For Cur@MSN, 15 mg of nanoparticles in 20 mL ethanol were mixed with 2 mL of curcumin-stock solution (1 mg/mL); for IR780@MSN, 15 mg of nanoparticles in 20 mL ethanol were mixed with 2 mL of IR780-stock solution (1 mg/mL); for Cur&IR780@MSN, encapsulating both anticancer agent and photosensitizer, 2 mL of curcumin-stock solution and 2 mL of IR780-stock solution were mixed in MSN solution. They were stirred in the dark and at room temperature for 24 h. Unloaded curcumin and/or IR780 were discarded with centrifugation. While the pellet was dissolved in 15 mL up-water (ultrapure water) for the following experiments, the supernatant was used to calculate the loaded amount of Cur and/or IR780.

### Surface Functionalization with PEG

To prevent early leakage and enhance the biocompatibility of nanoparticles, surface functionalization with polyethylene glycol (PEG-4000) was applied [32]. 15 mg of loaded MSN were stirred with 48 mg PEG inside water for 24 h at dark. Afterwards, the nanocarriers were washed three times.

### Characterization of Nanocarriers

The nanoparticle size distribution was determined with dynamic light scattering (DLS, Brookhaven Instruments 90 Plus) and scanning electron microscopy (SEM, Thermo scientific Quattro S). Thermo Scientific Nicolet 380 FTIR spectrometer was used to determine surface chemistry characteristics after each synthesis step. Absorption measurements were carried on via UV–VIS spectrophotometer (Thermo Scientific – Nanodrop 2000c).

### Laser Equipment and Procedure

In this research, we employed an infrared diode laser operating at 785 nm, coupled with a fiber and collimator system. The power intensity of the laser light was set at 500 mW/cm^2^ for all experiments. It's important to note that irradiation was conducted under dark conditions. The irradiation parameters presented in Table 1 were compiled using a combination of the manufacturer's specifications and calculated values derived from the measurements [33].

**Table 1:** Irradiation Parameters

Parameter [unit]	Value
Center wavelength [nm]:	785 ± 10
Spectral bandwidth [nm]:	31.54
Operating mode:	Continuous wave (CW)
Frequency [Hz]:	3.82 × 10^14^
Aperture diameter [μm]:	400
Beam divergence [mrad]:	< 3. 0
Beam profile:	Gaussian
Beam Spot Size [cm^2^]:	1.56
Irradiance at target [mW/cm^2^]:	500
Exposure duration [sec]:	300
Radiant exposure [J/cm^2^]:	150
Radiant energy [J]:	471
Area irradiated [cm^2^]:	3.14
Application technique:	Fiber Optic
Number and frequency of treatment sessions:	1

### Temperature Measurements

Cur&IR780@MSN were exposed to a 785-nm continuous laser (500 mW/cm^2^) with irradiation direction from top to the bottom of the cuvette to assess their photothermal effects in culture medium. Cur&IR780@MSN were added (500 µg/mL) to 1 mL culture medium and the temperature change was recorded under laser illumination. Also, the temperature change of IR780 alone was measured in the culture medium with laser exposure. The equal amount of culture medium exposed to the same laser irradiation served as the negative control. During the laser irradiation, temperature changes were monitored using an infrared camera (FLIR, E5-XT). The temperature and thermal images were recorded at 1 min intervals for a total of 5 min. Measurements were repeated three times.

### Reactive Oxygen Species Detection and Quantum Yield Determination

First, 10 mM DPBF was freshly prepared in 5 mL ethanol and then, 10 µL from the solution was mixed well with agents loaded MSN nanoparticles (1 mL, in PBS) for various concentrations: 0 µg/mL, 50 µg/ml, 100 µg/ml, 150 µg/ml, 300 µg/ml, 500 µg/ml. Next, 785 nm laser (500 mW/cm^2^) was applied for different time periods (0, 1, 2, 3, 4, and 5 min). The absorption intensity of the solution was recorded at 430 nm.

The quantum yield of Cur&IR780@MSN was calculated as defined by Ruhi et al*.* [18]. Methylene blue (MB) photosensitizer was utilized as the standard (quantum yield = 0.52). Both MB and nanocarriers were dispersed inside ethanol-DPBF solution. While MB was exposed to 660 nm wavelength (continuous, 100 mW/cm^2^), nanocarriers were irradiated with 785 nm laser light (continuous, 100 mW/cm^2^) to find out the rate of absorption change at 410 nm. Measurements were repeated three times.

### Curcumin Release

Curcumin release was determined as mentioned in literature [32]. PBS solution was prepared at two different pHs, 7.4 and 6.5. Nanocarriers with various concentrations waited inside PBS for 48 h at 100 RPM, 36 ˚C. Then, curcumin release was detected from the supernatant by using an absorption spectrophotometer.

### Cell Culture

RPMI-1640 medium having 10% fetal bovine serum and 1% penicillin/streptomycin solution was used to culture lung cancer cells (A549). Healthy lung cell lines (MRC5) were cultured using DMEM/F12 medium supplemented with 10% fetal bovine serum and 1% penicillin/streptomycin solution.

### Cellular Uptake of Nanocarriers

A549 was cultured on cell culture slides in a 6-well plate for 24 h until reaching 60–70% confluence. Then, cells were treated with 500 μg/mL of Cur&IR780@MSN for 2 h at 37 °C. After that, the culture medium was removed, and cells were rinsed with PBS 3 times and fixed with 4% paraformaldehyde solution at 37 °C for 15 min. DAPI was then used to stain the cells for 10 min at room temperature. A Leica SP5-AOBS confocal laser scanning microscopy (CLSM) with an × 40 water immersion lens and a resolution of 2.048 × 2.048 pixels was used to see the stained cells. With the aid of 405 and 488 nm diode lasers, DAPI and Cur&IR780@MSN were excited. The emission filter for DAPI ranges from 447 to 495 nm, while for Cur&IR780@MSN it ranges from 495 to 590 nm.

### *In vitro* Therapies

Cancer and healthy lung cell lines were placed in 96 and 24 well-plate at 8000 cells/well and 15,000 cells/well, respectively. After 24 h incubation for cellular adhesion, the cell medium was replaced with new media, including different concentrations of nanocarriers 50 µg/ml, 100 µg/ml, 150 µg/ml, 300 µg/ml, 500 µg/ml, and waited for 2 h. Subsequently, cells were rinsed with PBS thrice and introduced to a fresh cell medium.

The individual effects of MSN, IR780@MSN, and Cur@MSN were measured on only cancer cells; on the other hand, the efficiency of combined therapy (Cur&IR780@MSN) was determined on both cancer (A549) and healthy (MRC5) lung cells. During laser experiments, 785 nm wavelength of light was performed with 0.5 W/cm2 intensity for 5 min. The viability of cells was assessed with an MTT assay and microplate reader. The experiments were repeated three times.

### AO and PI Measurements

The effect of nanocarriers on cell viability was investigated with the acridine orange/propidium iodide (AO/PI) dual staining protocol [34]. Briefly, A549 cells were seeded into a 96-well plate and allowed to reach 80% confluence. Then, they were treated with 150 µg/ml of Cur@MSN, IR780@MSN, and Cur&IR780@MSN. Laser (785 nm, 0.5 W/cm2, 5 min) was employed for the IR780@MSN and Cur&IR780@MSN groups. Following 24-h incubation, PI and AO dyes were introduced to cells at the same time. The staining solution was withdrawn after a few minutes, and the cells were rinsed with PBS. Then, A549 cells were visualized with a fluorescent microscope.

### Detection of Singlet Oxygen with SOSG

SOSG solution was prepared according to the manufacturer’s suggested method. Shortly, 33 µL methanol was added to a 100 µg vial. Then, cellular media was used to obtain a 100 µM SOSG mixture. The freshly prepared solution was added to cells and waited for 20 min before applying the laser. Afterward, cells were cleaned with PBS thrice, and 785 nm laser illumination was introduced (500 mW/cm2, 5 min.); the cell images were taken immediately with fluorescence microscopy.

### Scratch Assay

Cellular migration of A549 was determined using a scratch assay [35]. Lung cancer cells were seeded (24 × 10^4^ cells/well) in a 24 well culture plate and incubated for 24 h. Once the cells’ confluency reached 100%, two straight lines were scratched with a 200 µL pipette tip. The wells were rinsed with PBS three times to remove the detached cells in media. Afterward, Cur&IR780@MSN at different concentrations (0, 50, 100, 150, 300, 500 µg/mL) were implemented and waited two hours for cellular uptake. While some groups were kept under dark conditions, PDT groups were subjected to a 785 nm laser (0.5 W/cm^2^, 5 min). The images of scratched parts were captured at 0 and 48 h. After repeating the experiments three times, migrated cells were quantified.

### Statistical Analysis

IBM SPSS Statistics program interface was used to calculate experimental groups’ significances between each other. Firstly, Shapiro Wilk test was implemented to decide whether the values were normally distributed or not. The normally distributed data sets were evaluated with one-way ANOVA (Analysis of Variance) and Tukey’s HSD (honestly significant difference) post hoc tests. The nonparametric values, on the other hand, were discussed with Kruskal Wallis statistics. Both *p < 0.05 and **p < 0.01 levels were calculated.

## Results

**Fig. 1:**
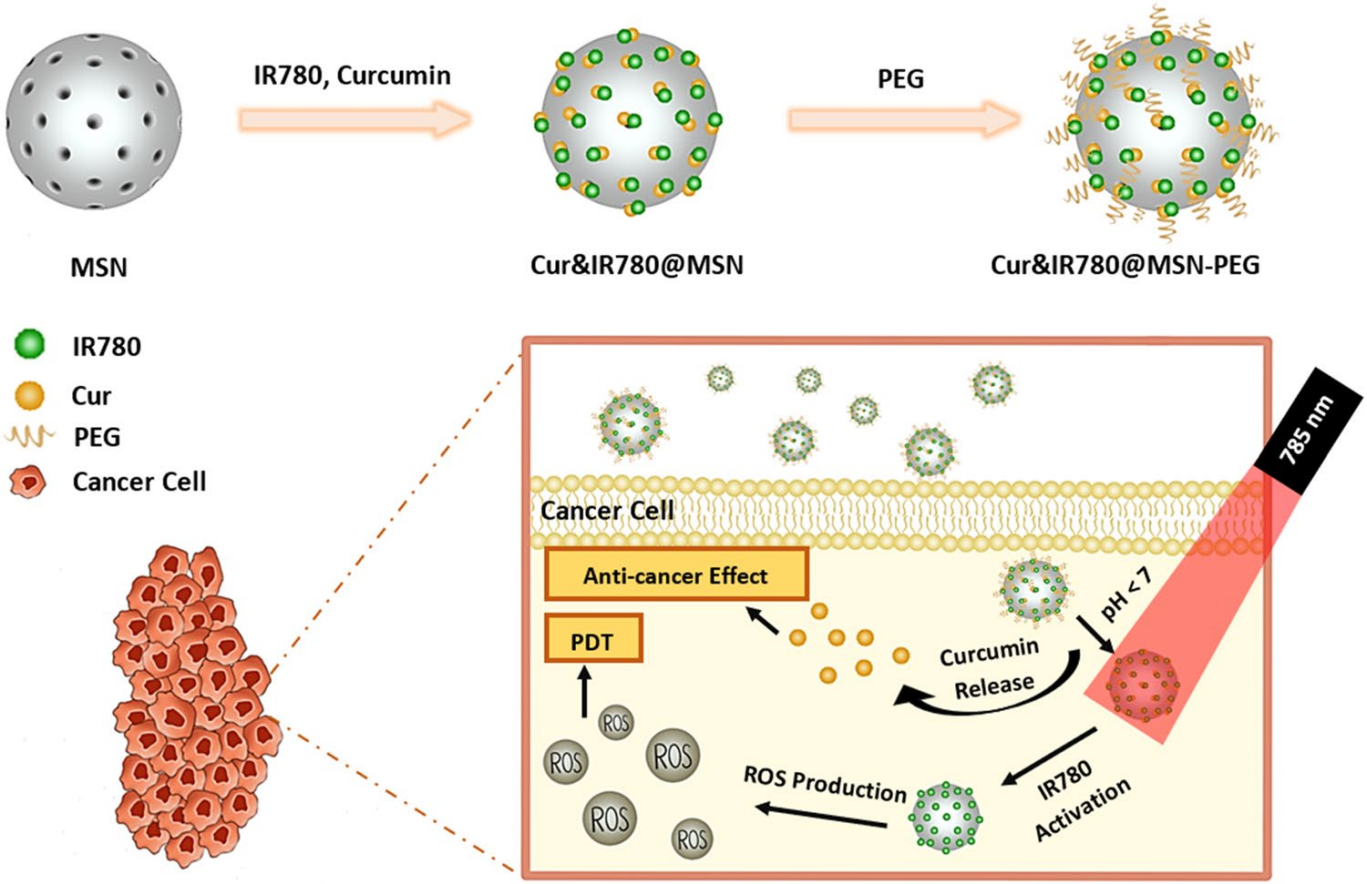
Schematic illustration of the nanocarriers synthesis, and combinational-therapeutic applications of Cur&IR780@MSN towards deep-seated tumors with NIR light

The nanocarriers, Cur&IR780@MSN-PEG, were produced within three main steps as illustrated in Fig. 1: (i) Mesoporous silica nanoparticles were synthesized, (ii) Curcumin and IR780 were loaded into MSN, and (iii) PEG was coated around the nanoparticles. These compact and easily fabricated carriers were aimed to introduce to the lung cancer cells. In this study, synergistic effect of near-infrared light activated photodynamic therapy and anti-cancer agent activity were investigated in detail to clear off lung cancer cells with minimizing the damage for healthy cells.

### Preparation and Characterization of Nanocarriers

**Fig. 2:**
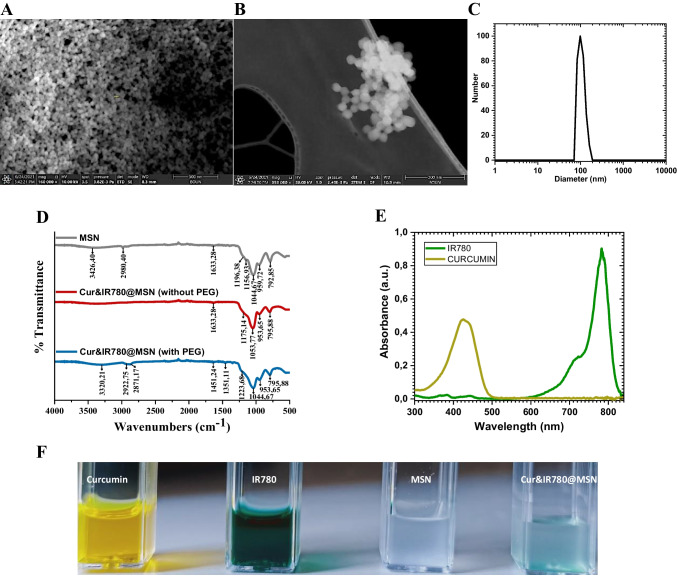
The synthesized porous silica nanoparticles (**A**) SEM images (500 nm scale), (**B**) STEM images (300 nm scale), and (**C**) hydrodynamic size (PDI: 0,111); (**D**) FTIR measurements during the production stages of nanocarriers, (**E**) absorption properties of IR780 and curcumin; and (**F**) digital photographs of Curcumin, IR780, mesoporous silica nanoparticles, and Cur&IR780@MSN

Stöber process was performed successfully to form mesoporous silica nanoparticles [31, 36, 37]. While TEOS was used as a silica source with a CTAB template, HCL etchant was employed to reveal pores. The size and homogenous distribution of nanoparticles were measured with SEM (equipped detector: STEM) and DLS. Both dry and solvent (in ethanol) MSN had a diameter close to 100 nm (Fig. 2A-C). Additionally, the monitored Fourier transform infrared spectrum of MSN demonstrated O–H stretching at 3426,40 cm^−1^ and Si–O bands between 1110 cm^−1^ – 830 cm^−1^ (Fig. 2D) [38]. The functional groups of Curcumin and IR780 were also detected as given in Figure S1. FTIR spectrum of curcumin showed the phenolic hydroxyl groups stretching around 3500,34 cm^−1^, C = C (alkenes) and carbonyl (C = O) groups’ vibrations at 1626,26 cm^−1^, and stretching of aromatic C = C groups at 1487,40 cm^−1^ [39, 40]. The outcome of IR780 with FTIR resulted in peaks between 1600 – 1430 cm^−1^ due to stretching of C = C and C = N rings [41]. When curcumin and IR780 were encapsulated in MSN, the characteristic behaviors of the curcumin and IR780 spectrum disappeared (Fig. 2D – red line). This means that both agents were loaded inside the pores. After PEG coating, the FTIR peaks of polymer appeared around 2922 cm^−1^ and 1044 cm^−1^ because of C-H and C-O stretching, respectively [42]. Meanwhile, the absorption peaks of curcumin at 430 nm and IR780 at 780 nm were detected as presented in Fig. 2E.

Figure 2F presents the digital images of curcumin, IR780, MSN, and Cur&IR780@MSN. Curcumin and IR780 were loaded into MSN by exploiting physical encapsulation. After loading curcumin and IR780 into MSN, nanocarriers were well dispersed inside PBS, as observed in Fig. 2F. The optical features of both agents were used to calculate the loaded amounts. Regarding the linear absorbance increase with respect to concentration rise (Figure S2-A and S2-B), the encapsulated Cur and IR780 into pores were determined with the conveyed relations (Eq. 1, Eq. 2 and Eq. 3) in the supplementary. The photosensitizers and anti-cancer agents were loaded together and separately to form three different nanocarriers; Cur@MSN, IR780@MSN, and Cur&IR780@MSN. The encapsulation efficiency and loading capacity for three different nanocarriers were calculated as given in Figure S2-C. Note that measurements were performed at least three times to exhibit the average data in Figure S2-C

**Fig. 3:**
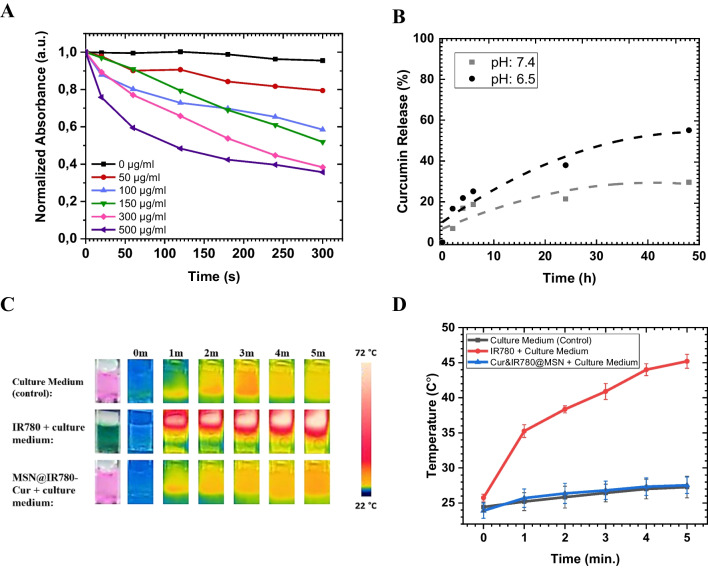
**A** DPBF bleaching of IR780 & Curcumin loaded MSN with different concentrations under 785 nm laser (500 mW/cm^2^) for 5 min duration, and **B** Curcumin release profile at different pH conditions. Temperature measurements for cellular culture medium, IR780, and Cur&IR780@MSN with laser (785 nm, 500 mW/cm^2^, 5 min) irradiation; **C** thermal camera images, **D** average temperature change

Although healthy tissues need reactive oxygen species (ROS) to retain their metabolic activities, excess amounts of ROS result in cellular death [43]. Photodynamic therapy, on the other hand, elevates the ROS level to wipe out cancerous tissue. This research aimed to apply PDT via encapsulating photosensitizers in nanoparticles. Cur&IR780@MSN's ability to generate ROS under 785 nm laser demonstrates the efficiency of PDT. A known ROS probe DPBF was mixed with the nanocarriers to detect the ROS level. Increasing concentrations of Cur&IR780@MSN from 0 µg/ml to 500 µg/ml resulted in DPBF bleaching, as demonstrated in Fig. 3A. The diminished absorbance of DPBF at 430 nm for all groups was significantly different from the control 0 µg/ml (p < 0.05). 50 µg/ml, 100 µg/ml -150 µg/ml, and 300 µg/ml – 500 µg/ml were reduced the DPBF solution absorbance approximately 20%, 40%, and 60% respectively, under 785 nm light radiance (500 mW/cm2) within 5 min. Along with the detected ROS, the singlet oxygen quantum yield of nanocarriers was estimated as 0.076 by using the obtained values in Figure S3.

Herein, the anti-cancer agent curcumin release rate was also determined concerning pH change. Compared to physiological conditions between the healthy and cancerous regions in the body, intratumoral compartments include a more acidic environment (pH 5.5–6.5) than other sites (pH 7.4–7.5) [44]. In this study, the designed nanocarriers were functionalized with polyethylene glycol to prevent early leakage of the agents and enhance biocompatibility. It is also known that PEG is one of the specific polymers for pH-sensitive drug release, as changes in pH values can alter the electrical properties of PEG. [45]. In Fig. 3B, the release of curcumin from Cur&IR780@MSN was presented for both pH 7.4 and 6.5. The follow-up experiments for these results included dissolving 1.5 mg/mL nanocarriers inside PBS (pH: 7.4 and 6.5) and shaking at 100 RPM for 48 h. Total curcumin release from nanocarriers over 48 h was observed as 25% and 55% at pH 7.4 and pH 6.5, respectively.

It has been shown that the presence of IR780 photosensitizers inside nanoparticles can activate photothermal therapy (PTT) alongside PDT [46]. To detect the PTT property of IR780 inside Cur&IR780@MSN (500 µg/mL concentration) under 785 nm laser irradiation, the temperature changes were measured by using thermal camera. As control group, NIR light was directly applied to cell culture media. When 1 mL culture medium exposed to 785 nm laser light (power: 500 mW/cm^2^, diameter of beam: 1 cm), a negligible temperature rise was recorded as 2 ºC in 5 min (Fig. 3C). Afterwards, only IR780 photosensitizer was mixed with cell culture medium. Since the estimated amount of IR780 in 500 µg of nanocarriers (Cur&IR780@MSN) was 1.87 µg (Table S1), cellular culture media was initially prepared with 1.87 μg/mL IR780 concentration. Laser irradiation was implemented, as explained in the laser equipment and procedure section. Thermal camera images showed that the solution temperature was altered from room temperature to 72ºC, but the average change was about 22ºC (Fig. 3). This means only-IR780 inside cell culture medium offers photothermal therapy by converting the NIR light to heat energy.

Subsequently, same measurements were performed for Cur&IR780@MSN. Culture media (1 mL) involving 500 µg/mL concentration of nanocarriers was exposed to 500 mW/cm^2^ intensity of light (785 nm, 5 min.). Unexpectedly, only 2 °C temperature change was measured as monitored in control groups. This outcome suggests that the mesoporous silica prevents the temperature rise of IR780. That is, the designed nanocarriers cannot introduce photothermal therapy.

### *In Vitro* Experiments

**Fig. 4:**
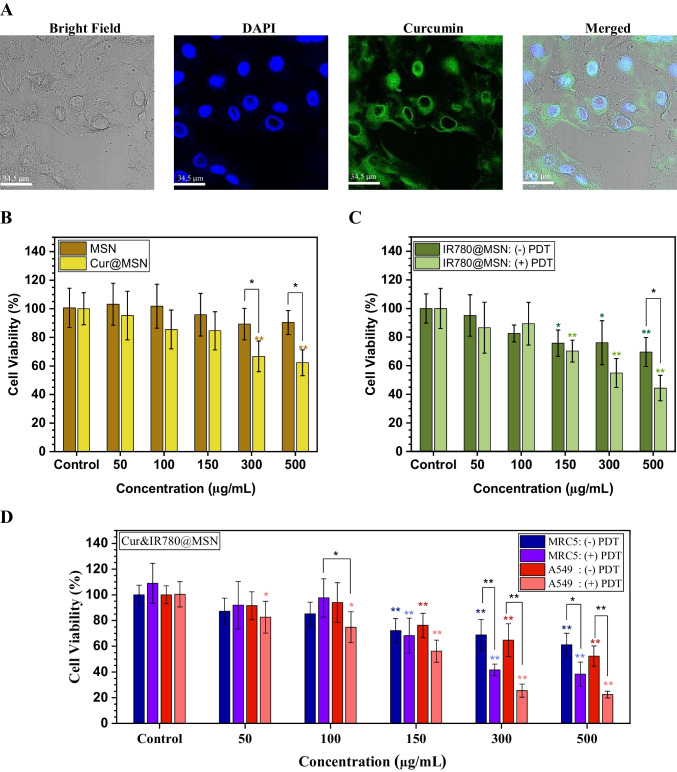
**A** Confocal microscope images showing cellular uptake of Cur&IR780@MSN in lung cancer cells (A549). The efficiency of nanocarriers on lung cancer cells, A549; **B** mesoporous silica cytotoxicity and curcumin encapsulated MSN analysis, **C** only IR780 loaded mesoporous silica (IR780@MSN) impact. **D** both curcumin—IR780 loaded mesoporous silica (Cur&IR780@MSN) activity under with/without laser irradiance (785 nm, 500 mW/cm^2^, 5 min) for cancer (A549) and healthy (MRC5) lung cells. (Significance marks directly above the bars indicate comparisons with control groups and top marks that follow show pairwise comparisons. ANOVA-Tukey (HSD) at *p < 0.05 and **p < 0.01 levels were used for all statistical analysis.)

Intracellular uptake of Cur&IR780@MSN was visualized by using CLSM as given in Fig. 4A. Before detecting the images, A549 lung cancer cells were incubated with nanocarriers for 2 h. Curcumin fluorescence signals showed that nanocarriers had gathered in the cytoplasm of the cells. In other words, Cur&IR780@MSN have been successfully taken up by the cells.

The MTT assay was used to decide the photodynamic and anti-cancer therapeutic effect of the engineered nanocarriers on lung cells. First, the necessity to observe the biocompatibility of PEG-coated MSN nanoparticles arose. Herein, biocompatibility refers to the minimal toxicity to cells against increasing material concentration without administering a laser [47]. No significant result was observed when lung carcinoma cells were exposed to different concentrations of MSN-PEG (Fig. 4B—(MSN)). The nanocarriers were highly biocompatible between 50 µg/mL and 500 µg/mL concentrations. Next, the anti-cancer effect of curcumin inside MSN was determined on cancer cells, as given in Fig. 4B—(Cur@MSN). Significant concentrations of Cur@MSN were found for the 300 µg/mL and 500 µg/mL groups. Both concentrations killed about 30% of cells upon curcumin leakage within 2 h.

To identify the efficiency of photodynamic therapy with IR780@MSN, PS encapsulated nanoparticles (IR780@MSN) were administered to lung cancer cells (Fig. 4C). Dark toxicity started at a concentration of 150 µg/mL. Between 150 – 500 µg/mL concentrations, the cell viability decreased by approximately 20 – 25%. For the experimental groups, 785 nm laser (500 mW/cm^2^, 5 min.) was applied to cells with IR780@MSN. PDT effect was significantly detected for pairwise group comparison only at 500 µg/mL, resulting in 50% of cancer cell death.

The data presented in Fig. 4D belong to cancer and healthy lung cells treated with Cur&IR780@MSN. For the (-) PDT groups, various concentrations of Cur&IR780@MSN were administered, and NIR laser was not used. Anti-cancer effect of curcumin and possible dark toxicity of IR780 on cancer cells can be observed in the A549: (-) PDT groups. Significance with Cur&IR780@MSN was detected at 150 µg/mL, and cell viability was around 50% for 500 µg/mL. On the contrary, 785 nm laser irradiated cancer cells (A549: ( +) PDT groups) showed significantly different results for all concentrations. That is, combination treatments were quite successful. According to pairwise comparisons with dark groups, significant results were acquired at 300 µg/mL and 500 µg/mL. The lowest cell viability (20%) was observed in the 500 µg/mL group.

The possible cytotoxicity of Cur&IR780@MSN on normal lung cells were also evaluated via direct application of photodynamic and anti-cancer therapies to MRC5 cell line, as given in Fig. 4D. Dark toxicity of nanocarriers became visible at 150 μg/mL, and cell viability was reduced by up to 60% with the highest concentration (500 μg/mL). When PDT was activated with laser (785 nm, 500 mW/cm2, 5 min) on healthy cells (MRC5: ( +) PDT groups), no significant result was observed until 150 μg/mL concentration. To add, the only significant difference between healthy and cancer cells was found in the 100 µg/mL group. At this concentration, 25% of cancer cells were killed, but 100% of healthy cells were alive for A549: ( +) PDT groups.

Along with MTT analysis, acridine orange/propidium iodide staining was utilized to determine cancer cell viability. This protocol shows alive and dead cells by vivid green and red fluorescence, respectively. Cur@MSN, IR780@MSN, and Cur&IR780@MSN possessing 150 µg/mL concentrations were subjected to cells. Their images were taken by fluorescence microscopy after laser irradiation to IR780@MSN and Cur&IR780@MSN. According to the given photographs in Figure S4, the untreated (control) cells had green and intact nuclei, indicating cells were alive. The cells turned red with the applied treatments. As hypothesized, the A549 cells treated with Cur&IR780@MSN had the most significant number of deaths.

The ROS production capability of Cur&IR780@MSN was formerly evaluated with the DPBF probe (Fig. 3A). Additionally, to observe singlet oxygen formation within the cells, singlet oxygen green (SOSG) probe was exploited just before 785 nm laser light illumination. SOSG interaction with singlet oxygen is well known to reveal SOSG endoperoxides. Thus, prevented electron transfers with the appearing endoperoxides result in green fluorescence around 525 nm [48]. SOSG measurements were obtained after applying Cur&IR780@MSN to lung cancer cells. The monitored images from the center of wells demonstrated that there is a concentration-dependent increase in green emissions (Figure S5). In other words, singlet oxygen levels were risen along with concentration.

**Fig. 5:**
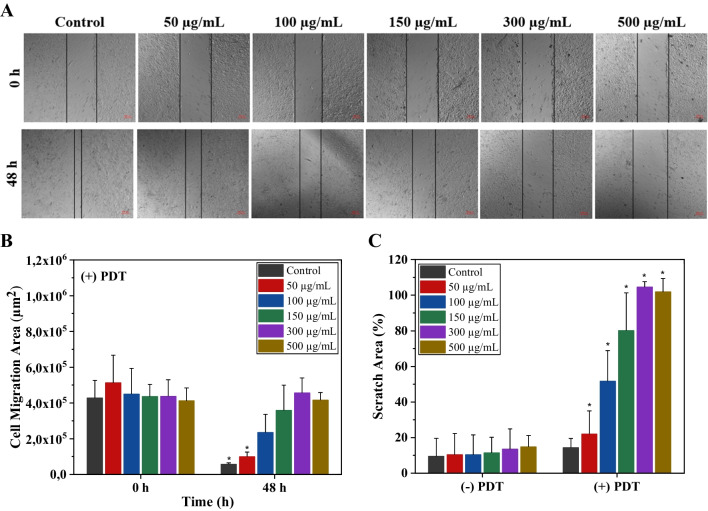
Cell scratch analysis of A549: (**A**) Bright-field microscope images (magnification: 5x) after application of Cur&IR780@MSN and 785 nm laser light (5 min., 500 mW/cm^2^), (**B**) The scratched cell migration area (μm.^2^) at 0 h and 48 h for different concentrations of nanocarriers (0 μg/mL, 50 μg/mL, 100 μg/mL, 150 μg/mL, 300 μg/mL, and 500 μg/mL), and (**C**) Relative percent of scratch area for both (-) PDT (without laser light) and ( +) PDT (with laser light). The presented values in the graphs stand for the mean and standard deviation of three replicates and the Kruskal Wallis nonparametric method was utilized to compare them (*P < 0.05)

Metastasis of cancer cells occurs due to migration and invasion of cells to distant sites in the body as the tumor grows. Scratch analysis was conducted to investigate the proposed PDT effect on cell migration. Initially, A549 cells were seeded inside 24 well-plate and waited till confluence was reached. Before applying the nanocarriers with various concentrations and exposing laser (500 mW/cm^2^, 5 min.), cells were scratched, and the residual was removed as described in the literature [49]. Images of A549 cells were taken at 0 h and 48 h with a microscope, as presented in Fig. 5A. The outcome demonstrated an excellent level of inhibition for cellular migration of the ( +) PDT groups. It was also observed that the concentration of nanocarriers had an impact on cell migration. After repeating the experiment three times, the cell migration area was calculated (Fig. 5B). The results showed that after 48 h, A549 cells could not manage to close the scratch significantly for the 100 μg/mL, 150 μg/mL, 300 μg/mL, and 500 μg/mL groups.

Furthermore, the same experiment was performed on lung cancer cells without laser application to detect cell migration behavior. It was observed that cells migrated in groups from the undamaged zone across the scratched regions (Figure S6). This demonstrated that the suggested nanoparticles had no significant impact on cell migration without laser irradiation. Herein, to quantify the cell (Fig. 5C), the area method was employed as explained in the supplementary (Eq. 5) [50]. Cancer cells closed the scratch area for all (-) PDT groups. Contrarily, the scratch area on the ( +) PDT groups showed no closure after 48 h, which means 5 min laser exposure prevented the proliferation of cells towards the scratched region (except 50 μg/mL group). This finding implies that the proposed novel nanocarriers containing curcumin and IR780 are highly efficient in preventing cancer cell migration.

## Discussion

Combined therapies for cancer treatment can give invaluable outcomes with an efficient nano-system design. The limitations of current cancer treatment modalities, such as serious damage to healthy tissue, can also be overcome; if alternative techniques, namely photodynamic or anti-cancer treatments, can be improved through nanotechnology. In this study, we utilized highly biocompatible silica and PEG materials to encapsulate hydrophobic agents (IR780 and Cur) by engineering a simple nano-system (Fig. 1). Lung cancer cells were used to determine the photodynamic or anti-cancer treatments efficacy with the developed nanocarrier.

Initially, mesoporous silica was produced with a newly-modified fast protocol via adjusting the chemical amount and temperature for the first time. Characterization results for MSN showed that they are homogeneous and well-dispersed particles (Fig. 2). Besides, they were preferred to be about 100 nm, as smaller sizes may pose higher toxicity, while larger sizes are often not well taken up by cells. The nanocarrier design also involved coating the silica with polyethylene glycol. To connect Si–O heads and PEG, the nanoparticles were directly mixed with the PEG at room temperature. FTIR spectra in Fig. 2D confirmed the successful junction.

The pore portions of the base nanocarriers were loaded with IR780 and curcumin. In order to evaluate the combined and individual behaviors of IR780 and Cur, three different nanocarriers were formed (Cur@MSN, IR780@MSN, and Cur&IR780@MSN), as given in the supporting information (Figure S2). Although using identical amounts of agents in individual loading experiments, it was observed that IR780 was entrapped to a greater extent than curcumin. This result may be due to the difference between the chemical structures of IR780 and curcumin. On the other hand, the simultaneous encapsulation of the two agents increased the loading capacity threefold. The similar result was detected in Author et al. (2021)’s previous research [38]. Based on these outcomes, it can be said that the drug affinity of silica pores enhances upon loading of two different agents at the same time.

The amount of ROS generated is highly crucial in PDT. Cur&IR780@MSN was evaluated for its ROS generation capacity outside the cells using the DPBF probe (Fig. 3A). Besides, the singlet oxygen production property of the nanocarriers was analyzed inside the cancer cells via the SOSG probe. Nanocarriers at different concentrations were exposed to laser, and the produced singlet oxygens were observed under fluorescence microscopy (Figure S5). The findings from DPBF and SOSG probes corroborated each other, showing a concentration-dependent increase in ROS generation. Furthermore, the release rate of anti-cancer agent, Cur, was also evaluated from Cur&IR780@MSN (Fig. 3B). It was observed that acidic conditions promoted a higher release of the agent compared to normal conditions. This effect may be attributed to the degradation of PEG linkages under acidic conditions [51].

PDT activation requires the application of light, which may cause an undesirable increase in temperature in the surrounding healthy tissue. When a 785 nm laser (500 mW/cm^2^, 5 min, continuous) was applied to the culture medium (without adding nanocarriers), the observed temperature increase was negligible (~ 2 °C) (Fig. 3C-3D). Additionally, the PTT ability of IR780 photosensitizer was studied. While alone-IR780 in culture medium resulted in temperature increase approximately 22 °C under 785 nm laser beam (500 mW/cm^2^) in 5 min, Cur&IR780@MSN inside culture medium did not show any temperature change upon NIR light application for the same time duration. This difference may be attributed to the small pore size of the silica, which restricts molecular vibrations that could otherwise lead to temperature increase. As a consequence, the designed Cur&IR780@MSN does not possess the ability to support PTT under NIR light. However, it is worth noting that widening the pore diameter of silica carriers can probably overcome this restrain. It should also be noted that employing PTT as a cancer treatment method can result in adverse effects. For instance, thermal stimulation can induce the excessive release of heat shock proteins [52]. This may result in the emergence of thermo-resistant cancer cells and tumor recurrence. In contrast, the proposed nanosystem in this study triggers PDT under near-infrared light with minimized risk of tumor recurrence as it does not activate PTT.

Valuable findings were obtained from conducting in vitro studies (Figs. 4 and 5). Initially, the effective accumulation of Cur&IR780@MSN within the cytoplasm of cancer cells was observed through the utilization of the fluorescence characteristic of curcumin. Then, dark toxicity of MSN (without agents loading) was evaluated. MSN at concentrations of 0 – 500 µg/mL did not exhibit detectable dark toxicity on cells, indicating a high level biocompatibility for MSN. When the anti-cancer treatment was applied using Cur@MSN nanocarriers at concentrations of 300—500 µg/mL, curcumin dispersed from the nanocarriers into the cell interior, resulting in a 30% reduction in cancer cell viability. These results suggest that the natural anti-cancer agent curcumin is not effective enough in cancer treatment. On the other hand, when the photodynamic therapy efficacy of IR780@MSN was investigated through laser interaction with lung cancer cells, two significant findings were observed: (1) IR780@MSN exhibited dark toxicity at a concentration of 150 µg/mL, and (2) at the highest applied concentration of 500 µg/mL, it reduced cell viability by 50%. Indeed, working with a NIR light-activated PS for achieving deep tissue penetration hinder the attainment of significant success in photodynamic therapy since their low quantum efficiency result in the insufficient levels of ROS generation.

Combining photodynamic and anti-cancer treatment modalities for lung cancer cells yielded more effective outcomes. Particularly, 300 µg/mL and 500 µg/mL concentrations of Cur&IR780@MSN in combination with NIR laser application decreased the viability of cancer cells around 80%. These findings were supported by data obtained from acridine orange and propidium iodide (PI) staining experiments, which aligned with the results of the MTT assay. Furthermore, these findings were compared with the responses of normal lung cells under identical experimental conditions. Both the dark toxicity of nanocarriers and the photodynamic therapy (PDT) toxicity of Cur&IR780@MSN with laser application exhibited a lesser decrease in cell viability in normal lung cells compared to cancerous cells. Additionally, migration of cancer cells was studied. Scratch assay experiment demonstrated a remarkable reduction in cell migration following laser irradiation on cancer cells treated with Cur&IR780@MSN (Fig. 5). These observations suggest that the proposed treatment has the potential to effectively inhibit cancer cell migration and potentially impede metastasis.

## Conclusion

This work evaluated the synergistic combination of IR780 and curcumin compounds within mesoporous silica nanocarriers for lung cancer cells. The key properties of Cur&IR780@MSN include (i) hydrophobic agents’ encapsulation capability, (ii) pH–dependent curcumin release, and (iii) a safer treatment modality under NIR light.

The use of this specially designed nanocarrier system, in combination with near-infrared light-activated photodynamic and anticancer therapies, resulted in a substantial decrease in the viability of lung cancer cells when compared to the effectiveness of each therapy used alone. Cur&IR780@MSN did not have the same detrimental effects on normal lung cells as cancer cells. Furthermore, a scratch assay was used to study the impact of Cur&IR780@MSN on cell migration, and the findings demonstrated that nanoparticles effectively inhibit metastasis. This research demonstrates that MSN serves as an effective carrier system when combined with IR780 and Cur, pointing to a bright future for in-vivo and clinical applications.

## Supplementary Information

Below is the link to the electronic supplementary material.

Supplementary file1 (DOCX 2508 KB)
