# Supplementary material for: Synergistic effect of mesoporous silica nanocarrier-assisted photodynamic therapy and anticancer agent activity on lung cancer cells
Source: Lasers Med Sci. 2024 Mar 16;39(1):91. doi: 10.1007/s10103-023-03969-x (PMC10942901; doi:10.1007/s10103-023-03969-x)
Supplement: Supplementary file 1 — Supplementary file1 (DOCX 2508 KB) [file 10103_2023_3969_MOESM1_ESM.docx]

**SUPPORTING INFORMATION**

**SYNERGISTIC EFFECT OF MESOPOROUS SILICA NANOCARRIER-ASSISTED PHOTODYNAMIC THERAPY AND ANTICANCER AGENT ACTIVITY ON LUNG CANCER CELLS**

|  |
| --- |
| **Figure S1**: FTIR results for curcumin and IR780. |

| **A**  **** | **B**  **** |
| --- | --- |
| **C**   \| **Nanocarriers** \| **Agents** \| **Encapsulation Efficiency (%)** \| **Loading Capacity (%)** \| \| --- \| --- \| --- \| --- \| \| Cur@MSN \| Curcumin \| 0.09 \| 0.01 \| \| IR780@MSN \| IR780 \| 0.83 \| 0.11 \| \| Cur&IR780@MSN \| Curcumin \| 2.67 \| 0.36 \| \| IR780 \| 2.80 \| 0.37 \| | |
| **Figure S2.** Absorption against concentration curves for (A) curcumin at 425 nm and (B) IR780 at 780 nm, and (C) EE (%) and LC (%) of Cur@MSN, IR780@MSN, Cur&IR780@MSN. | |

**Calculations for Loaded Agents into MSN:**

IR780 and curcumin were dissolved in ethanol with different concentrations. Then, the concentration against absorption curves were acquired at 780 nm (for IR780) and 425 nm (for curcumin) peak points with spectrophotometer (Thermo Scientific – Nanodrop 2000c). The preferred baseline during measurements was 550 nm since both agents’ absorption was equal “0 (a.u.)” at this wavelength. In order to decide the loaded amounts of Cur and/or IR780 into MSN, eq. 1 was used. Afterwards, encapsulation efficiency (EE) and loading capacity (LC) were calculated with eq. 2 and eq. 3.

| $Concentrations of agents$  $in begining$ | $-$ | $Concentrations of agents$  $in supernatant$ | $=Loaded Amounts of$  $Cur and/or IR780$ | **eq. 1** |
| --- | --- | --- | --- | --- |
| $EE \left( \% \right)=\frac{Weights of agents added-Weights of agents in supernatant}{Weights of agents added}x100$ | | | | **eq. 2** |
| $LC \left( \% \right)=\frac{Weights of agents added-Weights of agents in supernatant}{Weights of nanoparticles}x100$ | | | | **eq. 3** |

| **Table S1.** Corresponding amounts of agents inside Cur&IR780@MSN | | | |
| --- | --- | --- | --- |
| **Amounts of nanocarriers**  **(1 mL media)** |  | **Amounts of Curcumin** | **Amounts of IR780** |
| 500 µg |  | 1.78 µg | 1.87 µg |
| 300 µg |  | 1.06 µg | 1.12 µg |
| 150 µg |  | 0.53 µg | 0.56 µg |
| 100 µg |  | 0.36 µg | 0.37 µg |
| 50 µg |  | 0.18 µg | 0.19 µg |

**Singlet Oxygen Quantum Yield Estimation of Cur&IR780@MSN**

The absorption changes of methylene blue (reference) and nanocarriers (Cur&IR780@MSN) was measured as given in Figure S3. Quantum yield of nanocarriers was calculated based on equation 4:

| $\Phi_{\Delta}^{nanocarriers}=\Phi_{\Delta}^{ref} \frac{m^{nanocarriers}}{m^{ref}} \frac{I_{abs}^{ref}}{I_{abs}^{nanocarriers}}$ | **eq. 4** |
| --- | --- |

where $\Phi_{\Delta}$ is the singlet oxygen quantum yield, $m$ represents the slope, and $I$ shows the absorption correction factor ($I=1-{10}^{-OD} ,$OD: optical density) of nanocarriers and reference photosensitizer.

| **A**  **** | **B**  **** |
| --- | --- |
| **Figure S3:** DPBF bleaching at 410 nm in the presence of **A)** methylene blue and **B)** Cur&IR780@MSN under continuous 660 nm and 785 nm laser light luminescence (100 mW/cm^2^), respectively. | |

| **Control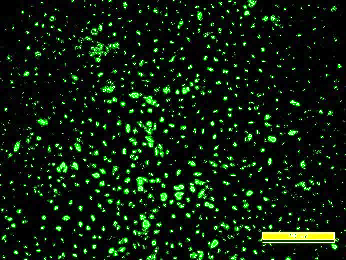** | **Cur@MSN**  **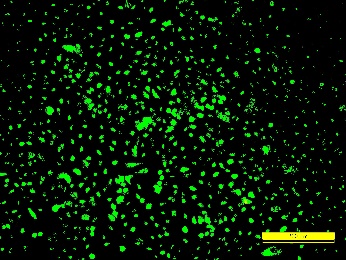** | **IR780@MSN: (+)PDT**  **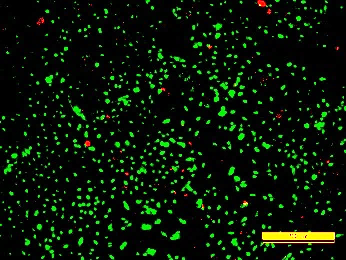** | **Cur&IR780@MSN: (+)PDT**  **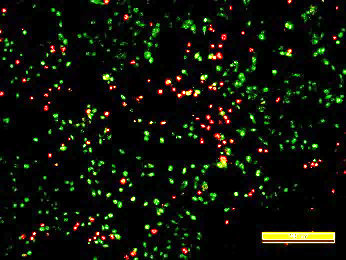** |
| --- | --- | --- | --- |
| **Figure S4:** Images of AO/PI staining for A549 upon 150 µg/mL nanocarriers implementation. | | | |

| \| **Control**  **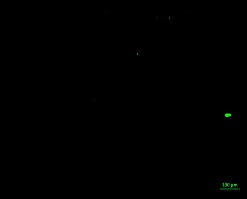** \| **50 µg/mL**  **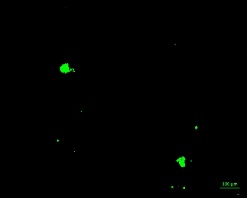** \| **100 µg/mL**  **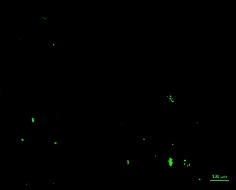** \| \| --- \| --- \| --- \| \| **150 µg/mL**  **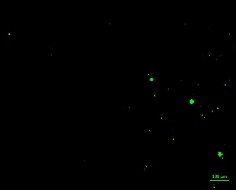** \| **300 µg/mL**  **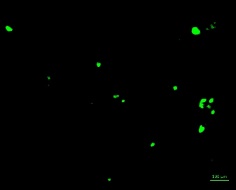** \| **500 µg/mL**  **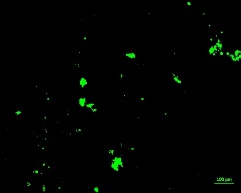** \| |
| --- | --- | --- | --- | --- | --- | --- |
| **Figure S5:** SOSG measurements for singlet oxygen detection upon 785 nm laser illumination (5 minutes, 500 mW/cm^2^). |

**Scratch Analysis of Cells in Dark Conditions and Calculations**

| **A**  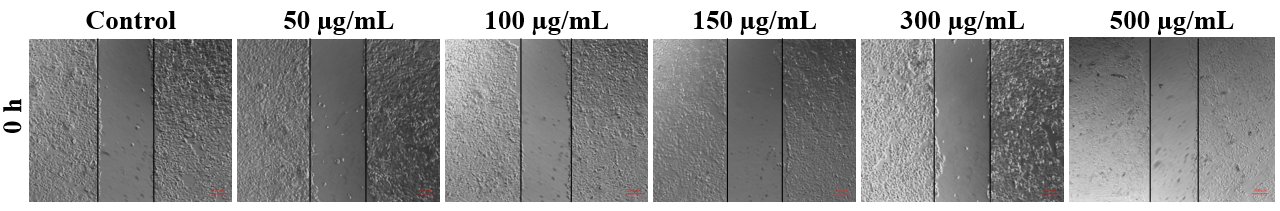 |
| --- |
| 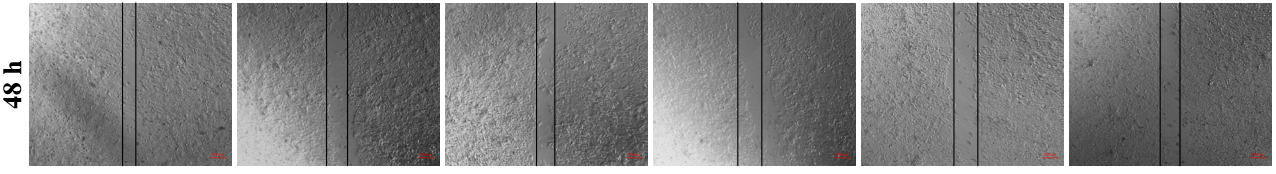 |
| **B**   |
| **Figure S6:** Scratch analysis of lung cancer cells under dark conditions; (A) microscope images, and (B) the calculated cell migration area (μm^2^). The presented values in the graphs stand for the mean and standard deviation of three replicates and Kruskal Wallis nonparametric method was utilized to compare them (*P<0.05). |

After the scratch experiments, the obtained images from the bright field of optical microscope were uploaded inside the imageJ program to give the cell migration area. The results were introduced in Figure S6 and Figure 5 as the dark and light implemented conditions, respectively. The drawn black line inside the figures indicate the evaluated area. Then, scratch area was calculated from the results. To assess the quantification, the eq. 5 was used.

| $A_{scratch\_area}= \frac{A_{48 h}}{A_{0 h}} x 100$ | **eq. 5** |
| --- | --- |

where $A_{scratch\_area}$ is the percentage of scratch area, $A_{48 h}$ and $A_{0 h}$ presents the cell migration areas at 48 h and 0 h, respectively.
